# Supplementary material for: Monocytic Differentiation in Acute Myeloid Leukemia Cells: Diagnostic Criteria, Biological Heterogeneity, Mitochondrial Metabolism, Resistance to and Induction by Targeted Therapies
Source: Int J Mol Sci. 2024 Jun 8;25(12):6356. doi: 10.3390/ijms25126356 (PMC11203697; doi:10.3390/ijms25126356)
Supplement: Supplementary file 1 [file ijms-25-06356-s001.zip › ijms-3020383-supplementary.pdf]

Monocytic differentiation in acute myeloid leukemia cells; diagnostic criteria, biological heterogeneity, mitochondrial metabolism, resistance to and induction by targeted therapies

Øystein Bruserud, Frode Selheim, Maria Hernandez-Valladares and Håkon Reikvam

Table S1. Molecular markers associated with FAB-M4/M5 differentiation of primary human AML cells [2,6,260-269]. The information is based on the Gene database (accessed November 31 2023) and selected references from the PubMed database.

Identity	Summary of important biological characteristics	Key words
CD4	*CD4 molecule.* This membrane glycoprotein acts as a coreceptor together with the T-cell antigen receptor in the context of class II MHC presentation, but it is also expressed by several other cells including macrophages where CD4 ligation triggers monocyte differentiation towards macrophages [260].	Cell surface<br>Macrophage differentiation
CD7	*CD7 molecule.* This transmembrane protein is a member of the immunoglobulin superfamily. CD7 ligation regulates monocyte migration by activating PI3K-Akt-mTOR signaling [261].	Monocyte migration<br>PI3K-Akt-mTOR
CD11B/ITGAM	*Integrin subunit alpha M.* Integrins are heterodimeric proteins composed of an alpha chain and a beta chain. The encoded integrin alpha M chain. combines with the beta 2 integrin chain (ITGB2) to form a leukocyte-specific integrin also referred to as macrophage receptor 1 ('Mac-1'), or inactivated-C3b (iC3b) receptor 3. The alpha M/beta 2 integrin is important in the adherence of neutrophils and monocytes to stimulated endothelium, and also for phagocytosis of complement-coated particles.	Integrin<br>Cell adhesion<br>Complement receptor<br>Phagocytosis
CD11C/ITGAX	*Integrin subunit alpha X.* The encoded integrin alpha X chain combines with the beta 2 chain (ITGB2, similar to CD11B/ITGAM, see above) to form a leukocyte-specific integrin referred to as inactivated-C3b (iC3b) receptor 4 (CR4). The alpha X beta 2 complex is important for the adherence of neutrophils and monocytes to stimulated endothelial cells.	Integrin<br>Cell adhesion<br>Complement receptor
CD13/ANPEP	*Alanyl aminopeptidase, membrane (aminopeptidase N).* This plasma membrane molecule has a extracellular carboxyterminal domain that contains a pentapeptide consensus sequence characteristic of members of the zinc-binding metalloproteinase superfamily. The enzyme is possibly involved in the metabolism of regulatory peptides by macrophages and granulocytes. This gene also seems to support angiogenesis and growth of malignant cells.	Metalloproteinase<br>Angiogenesis<br>Leukemogenesis
CD14	*CD14 molecule.* This cell surface protein is preferentially expressed on monocytes/macrophages. It is a coreceptor for Toll-like receptor 4 (TLR4), a cell surface receptor that can bind a wide range of endogenous ligands [262]. TLR4 ligation initiates NFκB activation that is important for the regulation of constitutive cytokine release by primary AML cells, and TLR responsiveness is associated with NPM1 mutations [263]. CD14 is also involved in the regulation of adhesion molecule expression and thereby the adhesion of AML cells cell to endothelial cells [264,265].	TLR4<br>Leukemogenesis<br>NFκB<br>Cytokine release<br>Cell adhesion
CD15/FUT4	*Fucosyltransferase 4.* The protein transfers fucose to N-acetyllactosamine polysaccharides to generate fucosylated carbohydrate structures. CD15 binds various selectin adhesion molecules and thereby mediates interactions/binding between leukocytes and endothelial cells (E-selectin, P-selectin) and various leukocytes (L-Selectin) [266].	Cell adhesion<br>Selectins<br>Endothelium
CD33	*CD33 molecule.* The encoded molecule has protein phosphatase and sialic acid binding activity and is a negative regulator of cytokine production and monocyte activation and a positive regulator of protein tyrosine phosphatase activity. It is located in several compartments including the Golgi apparatus, external side of the plasma membrane and peroxisomes. The siglecs (sialic acid-binding Ig-like lectins) are a family of transmembrane receptors. The CD33-related siglecs usually function as inhibitors of signaling mediated by receptors coupled to ITAMs (immunoreceptor tyrosine-based activation motifs) through recruitment and activation of SHP-1 [SH2 (Src homology 2) domain-containing phosphatase-1] and SHP-2, and they can suppress siglec-dependent adhesion of sialylated ligands and mediate endocytosis.	Protease binding<br>Sialic acid binding<br>Adhesion<br>Signaling
CD34	*CD34 molecule.* The encoded protein seems to have a role in the attachment of stem cells to the bone marrow extracellular matrix or to stromal cells. This membrane protein is highly glycosylated and phosphorylated by protein kinase C.	Stem cell adhesion<br>Extracellular matrix

CD36	*CD36 molecule.* The encoded protein serves as a receptor for thrombospondins that is an important adhesion molecule. It also binds to collagen, anionic phospholipids and oxidized low density lipoprotein. It binds long chain fatty acids and may function in the transport and/or as a regulator of fatty acid transport.	Thrombospondin<br>Cell adhesion<br>Collagen<br>Lipid metabolism
CD56/NCAM1	*Neural cell adhesion molecule 1.* The encoded member of the immunoglobulin superfamily is involved in cell-cell and cell-matrix interactions. It interacts with fibroblast growth factor receptors, N-cadherin and other components of the extracellular matrix, and it triggers signaling cascades involving FYN-focal adhesion kinase (FAK), mitogen-activated protein kinase (MAPK), and phosphatidylinositol 3-kinase (PI3K). Increased expression has been correlated with lower survival in AML.	Cell-cell adhesion<br>Cell-matrix<br>Intracellular signaling
CD63	*CD63 molecule.* The encoded protein is a member of the transmembrane 4 superfamily, also known as tetraspanins. Most of these proteins are cell-surface proteins that mediate signal transduction events and thereby regulate cell development, activation, growth and motility. The encoded protein is a cell surface glycoprotein known to complex with integrins.	Signal transduction<br>Integrin<br>Cell motility
CD64/FCGR1A	*Fc gamma receptor Ia.* This protein is a high-affinity Fc-gamma receptor.	Fc-γ receptor
CD65/VIM2	*CD65 molecule.* The CD65 protein is analogous to CD15 and is a type II chain fucoganglioside. It is possibly a CD62L/L-selectin ligand. It is normally expressed on a subset of monocytes and is expressed later than myeloperoxidase [267-269].	CD62L ligand
CD68	*CD68 molecule.* The encoded 110-kD transmembrane glycoprotein is a member of the lysosomal/endosomal-associated membrane glycoprotein family, and it localizes to lysosomes and endosomes with a smaller fraction circulating to the cell surface. The protein is a type I integral membrane protein with a heavily glycosylated extracellular domain and binds to tissue- and organ-specific lectins or selectins. It is also a member of the scavenger receptor family that function to clear cellular debris, promote phagocytosis, and mediate recruitment and activation of macrophages.	Lysosome/endosome<br>Lectins/selectins<br>Scavanger receptor
CD117	*KIT proto-oncogene, receptor tyrosine kinase.* This receptor tyrosine kinase is a glycosylated transmembrane protein with an N-terminal extracellular region with five immunoglobulin-like domains, a transmembrane region, and an intracellular tyrosine kinase domain at the C-terminus. Upon activation by stem cell factor the protein phosphorylates multiple intracellular proteins involved in regulation of proliferation, differentiation, migration and apoptosis. This protein has a soluble form.	Hematopoiesis<br>Stem cells
CD163	*CD163 molecule.* The encoded protein is a member of the scavenger receptor cysteine-rich superfamily and is exclusively expressed in monocytes and macrophages. It functions as an acute phase-regulated receptor involved in the clearance and endocytosis of hemoglobin/haptoglobin complexes, and may thereby protect tissues from free hemoglobin-mediated oxidative damage. This protein may also function as an innate immune sensor and as an inducer of local inflammation.	Scavanger receptor<br>Endocytosis
HLA-DR	*HLA-DRB1.* The HLA-DRB1 chain belongs to the HLA class II beta chain paralogs. This class II molecule is a heterodimer consisting of an alpha (DRA) and a beta chain (DRB), both anchored in the membrane. It presents antigenic peptides to CD4 T cells. Class II molecules are expressed in antigen presenting cells. Within the DR molecule the beta chain contains all the polymorphisms specifying the peptide binding.	Immunoregulation
LYSOZYME	*Lysozyme.* This gene encodes human lysozyme.	Antibacterial

Table S2. Classification of AML patients in high- and low-risk patients based on the RNA expression of five mitochondrial metabolism-related genes [71]; the table lists differentially expressed genes when comparing these two main subsets [262-265]. Molecules that are regarded as markers of FAB-M4/M5 differentiation (see Table S1), are marked by grey shadow. The information is based on the Gene database (accessed November 31 2023) and selected references from the PubMed database.

**Identity**	**Important biological characteristics**	**Key words**
AREG	*Amphiregulin.* The encoded member of the epidermal growth factor family is an autocrine growth factor as well as a mitogen for various cells, including fibroblasts. The protein interacts with the epidermal growth factor (EGF) and transforming growth factor alpha (TGF-alpha) receptor.	Autocrine growth<br>EGF<br>TGF
C5AR1	*Complement C5a receptor 1.* This protein enables G protein-coupled receptor activity and complement component C5a receptor activity. It is thereby involved in complement component C5a initiated signaling, including positive regulation of ERK1 and ERK2 cascade.	Complement<br>ERK1/2
CD14	*CD14 molecule.* The encoded protein is a surface antigen that is preferentially expressed on monocytes/macrophages. It is a coreceptor for Toll-like receptor 4 (TLR4), a cell surface receptor that can bind a wide range of endogenous ligands [262]. TLR4 ligation initiates NFκB activation that is important for the regulation of constitutive cytokine release by primary AML cells, and TLR responsiveness is associated with NPM1 mutations [263]. CD14 is also involved in the regulation of adhesion molecule expression and thereby the adhesion of AML cells cell to endothelial cells [264,265].	TLR4<br>Leukemogenesis<br>NFκB<br>Cytokine release<br>Monocyte marker
CD163	*CD163 molecule.* The encoded protein is a member of the scavenger receptor cysteine-rich (SRCR) superfamily, and is exclusively expressed in monocytes and macrophages. It functions as an acute phase-regulated receptor involved in the clearance and endocytosis of hemoglobin/haptoglobin complexes by macrophages, and may thereby protect tissues from free hemoglobin-mediated oxidative damage. This protein may also function as an innate immune sensor for bacteria and inducer of local inflammation.	Scavanger receptor<br>Endocytosis<br>Monocyte marker
CD1D	*CD1D molecule.* The encoded protein is a member of the CD1 family of transmembrane glycoproteins that are structurally related to the major histocompatibility complex (MHC) proteins and form heterodimers with beta-2-microglobulin. The CD1 proteins mediate the presentation of primarily lipid and glycolipid antigens of self or microbial origin to T cells. The protein encoded by this gene localizes to late endosomes and lysosomes via a tyrosine-based motif in the cytoplasmic tail.	Beta-2-microglobulin<br>Antigen presentation<br>Endosome<br>Lysosome
CD36	*CD36 molecule.* The encoded protein serves as a receptor for thrombospondin. Since thrombospondins are widely distributed proteins involved in a variety of adhesive processes, this protein may have important functions as a cell adhesion molecule. It binds to collagen, thrombospondin, anionic phospholipids and oxidized LDL (low density lipoprotein). It also binds long chain fatty acids and may function in the transport and/or as a regulator of fatty acid transport.	Thrombospondin<br>Cell adhesion<br>Collagen<br>Fatty acid metabolism
CD74	*CD74 molecule.* The encoded protein associates with class II major histocompatibility complex (MHC) and is an important chaperone that regulates antigen presentation for immune response. It also serves as cell surface receptor for the cytokine macrophage migration inhibitory factor (MIF) which initiates survival pathways and cell proliferation.	HLA class II<br>Antigen presentation<br>MIF receptor
CD96	*CD96 molecule.* The protein belongs to the immunoglobulin superfamily and is a type I membrane protein. The protein may have a role in cell adhesion and/or antigen presentation.	Adhesion<br>Antigen presentation
CYTL1	*Cytokine like 1.* This cytokine-like protein is specifically expressed in bone marrow and cord blood mononuclear cells that bear the CD34 surface marker.	Cytokine

FCER1A	*Fc epsilon receptor Ia.* The immunoglobulin epsilon receptor (IgE receptor) is the initiator of the allergic response. When two or more high-affinity IgE receptors are brought together by allergen-bound IgE molecules, mediators that are responsible for allergy symptoms are released. This receptor is comprised of an alpha subunit, a beta subunit, and two gamma subunits. The protein encoded by this gene represents the alpha subunit.	IgE Fc receptor
FGR	*FGR proto-oncogene, Src family tyrosine kinase.* This protein is a member of the Src family of protein tyrosine kinases (PTKs). The protein contains N-terminal sites for myristylation and palmitylation, a PTK domain, and SH2 and SH3 domains that are involved in protein-protein interactions with phosphotyrosine-containing and proline-rich motifs, respectively. The protein localizes to plasma membrane ruffles, and functions as a negative regulator of cell migration and adhesion triggered by the beta-2 integrin signal transduction pathway.	Tyrosine kinase<br>B2 integrin<br>Cell migration
GABRE	*Gamma-aminobutyric acid type A receptor subunit epsilon.* The encoded protein belongs to the ligand-gated ionic channel (TC 1.A.9) family. The gene encodes the gamma-aminobutyric acid (GABA) A receptor which is a multisubunit chloride channel; the gene encoding an epsilon subunit.	Ionic channel<br>Cell surface receptor
HK3	*Hexokinase 3.* Hexokinases phosphorylate glucose to produce glucose-6-phosphate, the first step in most glucose metabolism pathways. This gene encodes hexokinase 3. Similar to hexokinases 1 and 2, this allosteric enzyme is inhibited by its product glucose-6-phosphate.	Glucose metabolism
HLA-DPA1<br>HLA-DPB1<br>HLA-DRA<br>HLA-DRB1<br>HLA-DRB5	The genes encode HLA class II α and β chains for HLA-DR and HLA-DP molecules that are important for antigenic presentation/immunoregulation	Antigen presentation
HMOX1	*Heme oxygenase 1.* Heme oxygenase cleaves heme to form biliverdin, which is subsequently converted to bilirubin by biliverdin reductase. Heme oxygenase activity is induced by its substrate heme and by various nonheme substances. Heme oxygenase occurs as 2 isozymes, an inducible heme oxygenase-1 and a constitutive heme oxygenase-2.	Heme cleavage
IFITM3	*Interferon induced transmembrane protein 3.* Interferon-induced transmembrane (IFITM) proteins are a family of five members, including IFITM1, IFITM2 and IFITM3 and belong to the CD225 superfamily.	Interferon
LILRA5	*Leukocyte immunoglobulin like receptor A5.* The encoded protein is a member of the leukocyte immunoglobulin-like receptor (LIR) family. LIR family members have activating and inhibitory functions in leukocytes. Crosslink of this receptor protein on the surface of monocytes induces calcium flux and secretion of several proinflammatory cytokines, which suggests the roles of this protein in triggering innate immune responses.	Monocytes<br>Calcium flux<br>Proinflammatory<br>Cytokine release
LILRB2	*Leukocyte immunoglobulin like receptor B2.* The encoded protein is a member of the leukocyte immunoglobulin-like receptor (LIR) family. It belongs to the subfamily B class (see above LILRA5). This receptor is expressed on immune cells where it binds to MHC class I molecules on antigen-presenting cells and transduces a negative signal that inhibits stimulation of an immune response.	HLA class I<br>HLA binding<br>Immunosuppression
LILRB4	*Leukocyte immunoglobulin like receptor B4.* The encoded protein is a member of the leukocyte immunoglobulin-like receptor (LIR) family. It belongs to the subfamily B class of LIR receptors which contain two or four extracellular immunoglobulin domains, a transmembrane domain, and two to four cytoplasmic immunoreceptor tyrosine-based inhibitory motifs (ITIMs). The receptor is expressed on immune cells where it binds to MHC class I molecules on antigen-presenting cells and transduces a negative signal. The receptor can also function in antigen capture and presentation.	HLA class I<br>HLA binding<br>Immunosuppression<br>Antigen presentation

LMNA	*Lamin A/C.* The encoded protein is part of the nuclear lamina, a two-dimensional matrix of proteins located next to the inner nuclear membrane. The lamin family of proteins makes up the matrix. During mitosis, the lamina matrix is reversibly disassembled as the lamin proteins are phosphorylated. Lamin proteins are thought to be involved in nuclear stability, chromatin structure and gene expression.	Nucleus<br>Chromatin structure<br>Gene expression
LRP1	*LDL receptor related protein 1.* This protein is a member of the low-density lipoprotein receptor family. The encoded preproprotein is proteolytically processed by furin to generate 515 kDa and 85 kDa subunits that form the mature receptor. This receptor is involved in several cellular processes, including intracellular signaling, lipid homeostasis, and clearance of apoptotic cells.	LDL receptor
LRRC25	*Leucine rich repeat containing 25.* This membrane protein is predicted to be located in cytoplasm.	Membrane protein
MAFB	*MAF bZIP transcription factor B.* The protein is a basic leucine zipper (bZIP) transcription factor that plays an important role in the regulation of lineage-specific hematopoiesis. The encoded nuclear protein represses ETS1-mediated transcription of erythroid-specific genes in myeloid cells.	Hematopoiesis<br>Transcription
MPEG1	*Macrophage expressed 1.* The protein is involved in defense responses to bacteria. It is located in cytoplasmic vesicle.	Cytoplasm
MS4A6A	*Membrane spanning 4-domains A6A.* This protein is a member of the membrane-spanning 4A gene family that is characterized by common structural features and similar intron/exon splice boundaries and display unique expression patterns among hematopoietic cells and nonlymphoid tissues.	Hematopoiesis
NTNG2	*Netrin G2.* Predicted to be involved in several processes, including basement membrane assembly; cell morphogenesis involved in differentiation; and regulation of cell projection organization. It is located in Flemming bodies, intercellular bridges and plasma membrane.	Plasma membrane<br>Basement membrane
RFX8	*Regulatory factor X8.* Predicted to enable DNA-binding transcription factor activity, RNA polymerase II-specific and RNA polymerase II cis-regulatory region sequence-specific DNA binding activity. Predicted to be involved in regulation of transcription by RNA polymerase II. Predicted to be located in nucleus. Predicted to be part of chromatin.	Transcription<br>Chromatin<br>Nucleus
S100A6	*S100 calcium binding protein A6.* The protein is a member of the S100 family of proteins containing 2 EF-hand calcium-binding motifs. S100 proteins are localized in the cytoplasm and/or nucleus, and involved in the regulation of a number of cellular processes such as cell cycle progression and differentiation. S100 genes include at least 13 members which are located as a cluster on chromosome 1q21. This protein may function in stimulation of exocytosis.	Nucleus<br>Cytoplasm<br>Exosomes<br>Cell cycle
S100A8	*S100 calcium binding protein A8.* The protein is a member of the S100 family of proteins containing 2 EF-hand calcium-binding motifs. S100 proteins are localized in the cytoplasm and/or nucleus, and involved in the regulation of a number of cellular processes such as cell cycle progression and differentiation. This protein may function in the inhibition of casein kinase and as a cytokine.	Nucleus<br>Cytoplasm<br>Cell cycle<br>Cytokine
S100A9	*S100 calcium binding protein A9.* The protein is a member of the S100 family of proteins containing 2 EF-hand calcium-binding motifs. S100 proteins are localized in the cytoplasm and/or nucleus and are involved in the regulation of a number of cellular processes such as cell cycle progression and differentiation. This protein may function in the inhibition of casein kinase	Nucleus<br>Cytoplasm<br>Cell cycle
SLC11A1	*Solute carrier family 11 member 1.* This member of the solute carrier family 11 (proton-coupled divalent metal ion transporters) family is a multi-pass membrane protein. The protein functions as a divalent transition metal (iron and manganese) transporter involved in iron metabolism and host resistance to certain pathogens.	Solute carrier<br>Divalent ions<br>Iron metabolism
SORT	*Spectrin repeat containing, nuclear envelope 1.* This protein enables actin filament binding activity and protein homodimerization activity. Involved in centrosome localization and regulation of cilium assembly. It acts upstream of or within nuclear migration and	Nuclear envelope<br>Centrosome

	is located in the nuclear envelope.	
TNFRSF1B	*TNF receptor superfamily member 1B.* The encoded protein is a member of the TNF-receptor superfamily. This protein and TNF-receptor 1 form a heterocomplex that mediates the recruitment of two anti-apoptotic proteins, c-IAP1 and c-IAP2, which possess E3 ubiquitin ligase activity. The function of IAPs in TNF-receptor signaling is unknown, however, c-IAP1 is thought to potentiate TNF-induced apoptosis by the ubiquitination and degradation of TNF-receptor-associated factor 2, which mediates anti-apoptotic signals.	TNF<br>Apoptosis
VNN1	*Vanin 1.* This protein belongs to the vanin family that includes secreted and membrane-associated proteins, a few of which have been reported to participate in hematopoietic cell trafficking. The vanin proteins possess pantetheinase activity, which may play a role in oxidative-stress responses. This protein is likely a GPI-anchored cell surface molecule.	Oxidative stress<br>Cell trafficking<br>Oxidative stress
